# Serum Alpha‐Synuclein and Inflammatory Markers profile in an Egyptian Alzheimer’s and Parkinson’s Diseases patients: A Pilot Study

**DOI:** 10.1002/alz.093274

**Published:** 2025-01-09

**Authors:** Shimaa Adel Heikal

**Affiliations:** ^1^ The American University in Cairo, Cairo Egypt

## Abstract

**Background:**

Alzheimer’s and Parkinson's disease are the most common neurodegenerative diseases. In the current study, we explored the potential of blood‐based markers to differentiate Alzheimer's disease (AD) and Parkinson's disease (PD) from healthy controls using ELISA assays via measuring the serum level of α‐Syn and panels of inflammatory cytokines in the small pilot cohort of Egyptian volunteers. With the ongoing genetic studies, upcoming data suggest that it is not trivial to revisit the findings reported in specific populations to be tested in each ancestor of different genetic and environmental backgrounds.

**Method:**

A total of 42 participants were recruited from the Neurology department, Suhag University Hospital, including 18 AD cases, 9 PD cases, and 15 healthy controls. Clinical and demographic characteristics were well‐matched among the three groups.

**Result:**

The current data is the first to provide evidence in an Egyptian cohort that aligns with earlier reports that serum level of α‐synuclein can be a specific marker for distinguishing PD patients from healthy individuals but not AD patients. Both AD and PD, however, exhibited shared neuroinflammatory profiles with elevated IL‐6 and decreased IL‐10, hinting at a common inflammatory component despite their distinct etiologies. While trends toward increased IL‐1β and TNF‐α were observed in AD, the lack of statistical significance suggests a more limited role in its pathogenesis or the need to expand the sample size.

**Conclusion:**

These findings, although promising and the first of a kind to be conducted in Egyptian patients, necessitate further investigation with larger sample sizes to solidify these markers' potential for diagnosis and fully unravel the specific roles of individual cytokines in each disease.

